# Rare tradition of the folk medicinal use of *Aconitum* spp. is kept alive in Solčavsko, Slovenia

**DOI:** 10.1186/s13002-017-0171-x

**Published:** 2017-08-08

**Authors:** Marija Povšnar, Gordana Koželj, Samo Kreft, Mateja Lumpert

**Affiliations:** 1 Faculty of Pharmacy, University of Ljubljana, Aškerčeva cesta 7, -1000 Ljubljana, SI Slovenia; 2 Institute of Forensic Medicine, Faculty of Medicine, University of Ljubljana, Korytkova 2, -1000 Ljubljana, SI Slovenia

**Keywords:** Aconite, Monkshood, Ethnomedicine Balkan

## Abstract

**Background:**

*Aconitum* species are poisonous plants that have been used in Western medicine for centuries. In the nineteenth century, these plants were part of official and folk medicine in the Slovenian territory. According to current ethnobotanical studies, folk use of *Aconitum* species is rarely reported in Europe. The purpose of this study was to research the folk medicinal use of *Aconitum* species in Solčavsko, Slovenia; to collect recipes for the preparation of *Aconitum* spp., indications for use, and dosing; and to investigate whether the folk use of aconite was connected to poisoning incidents.

**Methods:**

In Solčavsko, a remote alpine area in northern Slovenia, we performed semi-structured interviews with 19 informants in Solčavsko, 3 informants in Luče, and two retired physicians who worked in that area. Three samples of homemade ethanolic extracts were obtained from informants, and the concentration of aconitine was measured. In addition, four extracts were prepared according to reported recipes.

**Results:**

All 22 informants knew of *Aconitum* spp. and their therapeutic use, and 5 of them provided a detailed description of the preparation and use of “voukuc”, an ethanolic extract made from aconite roots. Seven informants were unable to describe the preparation in detail, since they knew of the extract only from the narration of others or they remembered it from childhood. Most likely, the roots of *Aconitum tauricum* and *Aconitum napellus* were used for the preparation of the extract, and the solvent was homemade spirits. Four informants kept the extract at home; two extracts were prepared recently (1998 and 2015). Three extracts were analyzed, and 2 contained aconitine. Informants reported many indications for the use of the extract; it was used internally and, in some cases, externally as well. The extract was also used in animals. The extract was measured in drops, but the number of drops differed among the informants. The informants reported nine poisonings with *Aconitum* spp., but none of them occurred as a result of medicinal use of the extract.

**Conclusions:**

In this study, we determined that folk knowledge of the medicinal use of *Aconitum* spp. is still present in Solčavsko, but *Aconitum* preparations are used only infrequently.

## Background

**Fig. 1:**
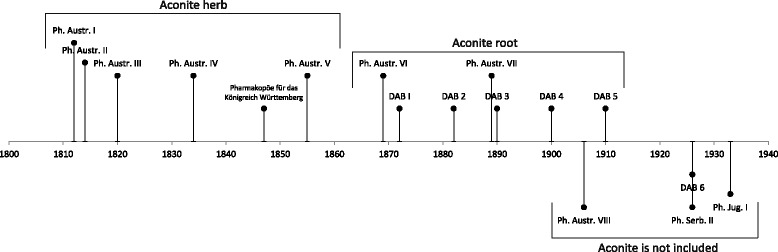
Timeline of use of aconite species (*Aconitum* spp.) in German pharmacopoeias and official pharmacopoeias of the Slovenian territory in the nineteenth century and in the beginning of the twentieth century

*Aconitum* species belong to the Ranunculaceae family, and they have been used in both Western and Eastern medicine for centuries [1]. This genus is still used as home medicine and food in central China [2]. The Austrian Pharmacopeia, *Pharmacopoeia Austriaca* (Ph. Austr.), was the official pharmacopoeia of the Slovenian territory in the nineteenth century [3]. A monograph entitled *Aconitum* (or *Herba Aconiti*) and a recipe for the extract of aconite herb was included in the first five editions of Ph. Austr. (1812, 1814, 1820, 1834, and 1855) (Fig. 1) [4–8]. The sixth and seventh editions of Ph. Austr. (1869 and 1889) contained a monograph on aconite root (Radix Aconiti) [9, 10]. The monograph specified that the roots of *Aconitum napellus* Linn., *Aconitum neomontanum* Wulf., and *Aconitum tauricum* Wulf. can be used for the preparation of aconite extract and aconite tincture. Aconite was not included in the eighth edition of Ph. Austr. (1906) [11], which was the official pharmacopoeia of the Slovenian territory until 1926 [3]. Between 1926 and 1934, the second edition of the Serbian Pharmacopeia, *Pharmacopoeia serbica editio secunda* (1908), was the official pharmacopoeia of the Slovenian territory [3]; it did not contain aconite [12]. Similarly, no monograph or recipe for aconite was included in the first Yugoslavian Pharmacopeia, *Pharmacopoeia jugoslavica* (1933), which became the official pharmacopoeia of the Slovenian territory in 1935. However, toxic and lethal doses of aconitine (Aconitinum) and aconite root (Aconiti tuber) were provided; in addition, the symptoms and treatment of intoxication with aconitine were described [13].

Similar to the first five editions of the Ph. Austr., the Pharmacopoeia for the Kingdom of Württemberg, *Pharmakopöe für das Königreich Württemberg* (1847), also described aconite herb in a monograph entitled *Herba Aconiti* and included a recipe for aconite tincture [14]. Furthermore, similar to the sixth and the seventh editions of Ph. Austr. (1869, 1889), the first five editions of the German Pharmacopoeia, *Deutsches Arzneibuch* (DAB) (1872, 1882, 1890, 1900, and 1910), contained a monograph on aconite root (Tubera Aconiti) and a recipe for aconite tincture (Tinctura Aconiti) [15–19]. The sixth edition of DAB (1926) no longer contained a monograph on aconite [20].

Medicinal use of aconite roots was described in the German handbook of general and special prescriptions for doctors, *Handbuch der allgemeinen und speziellen Arzneiverordnungslehre für Ärzte* (1929) [21]. Extract of aconite roots was used internally for rheumatism and gout; it was prepared as pills or drops for oral administration. Tincture of aconite roots was used internally for neuralgia. The extract was also used externally (in liniments for rubbing). The handbook also mentioned the internal use of aconite herb prepared in powders and pills and used for rheumatism, gout, and neuralgia in France. A Slovenian pharmacognosist, Pavle Bohinc, described the medicinal use of aconite in a book about the cultural history of natural ingredients (1992). Aconite extract was used externally for trigeminal neuralgia, lumbago, sciatica, arthritis, gout, and rheumatism because of its anesthetic properties. It was also used in a liquid formulation for rinsing and gargling for the treatment of colds, sore throats and inflammation of the respiratory tract. Doctors also prescribed aconite extract internally to treat headache, toothache, cough, and fever [22].

In Slovenia, the aconite plant was also familiar to many ordinary people. Möderndorfer (1964) reported that aconite root had been built into feed troughs for pigs and was also used as folk medicine for people with toothaches and snake bites in the beginning of the twentieth century [23]. Makarovič (2001) reported that an infusion of aconite roots was used internally to treat stomachaches and spasms in the middle of the twentieth century [24]. In a few newspaper advertisements (from 1919, 1939, 1950, and 1966), it is evident that cooperatives, pharmacies, and pharmaceutical companies encouraged readers to collect aconite roots in exchange for money [25–29]. Two articles from 1935 and 1938 describe the medicinal use of aconite but warn readers that the plant is very poisonous and should not be used as a homemade medicine [30, 31]. In 1892, Martin Cilenšek, a Slovenian botanist, described the plant *Aconitum Napéllus* in a popular scientific book about poisonous plants; he also warned the readers of its poisons, listed symptoms of poisoning and described six cases of poisoning with different parts of the plant [32]. Aconite was not mentioned in the most popular books about medicinal plants in Slovenia, which were authored by Father Simon Ašič [33–35] and Marija Treben [36], and it was rarely mentioned in other books about medicinal plants. Two books by foreign authors mentioned its homeopathic use [37, 38], and one of them discussed its external use for the treatment of pain due to contusions, sciatica, rheumatism, and neuralgia [37].

According to national legislation, the medicinal use of *Aconitum* species is not allowed in Slovenia due to their toxicity [39]. *Aconitum* species contain many alkaloids (aconitine, mesaconitine, and hypoaconitine). The concentration of alkaloids varies among species and also depends on the plant’s origin, time of harvest, and processing procedures. Alkaloids may cause cardiac arrhythmia, hypotension, gastrointestinal upset, and neurologic symptoms (numbness of the mouth and paresthesia in the extremities) [1].

According to the above-mentioned Austrian pharmacopoeias, aconite was part of the official medicine in nineteenth century Slovenia. However, the rural population used professional health services infrequently in the nineteenth century due to the lack of physicians, lack of material resources, mistrust of physicians and medicines, and language barriers. During illness, people treated themselves or obtained help from a folk healer, who treated them with herbs or magic practices [40]. In the early eighteenth century, Austrian laws forced healers to submit for approval a manuscript on which they relied for treatments. During that time, many translations and transcriptions of Renaissance books on medicinal plants were created [22]. Folk medicine manuscripts were translations and adaptations of mostly German medicine books, especially herbals (or Kräuterbücher) from the sixteenth century and the beginning of the seventeenth century [41].

One folk healer was Vid Strgar-Fida (1836–1922), who lived in Solčava, a village in northern Slovenia close to the Austrian border, and wrote a manuscript about medicinal plants titled “*Zdravje bolnikov to je bukve polne navkov za človeško zdravje spisano k pomoči kmetiškim ljudem v Sulcbahi 1866*” (in English: Health of patients - this is a book full of knowledge about human health written to help peasant people in Sulcbah 1866). In the preface, Vid Strgar wrote that the book was based on the book by Nicolas Lémery, a doctor in a pharmacy in Paris. Vid Strgar included a chapter about poisonous plants, “*Strupene Rože*”. The seventh plant he described was aconite, which at that time was called “lesjak” in Slovene; he also gave its German name, “eisenhutkraut”. He wrote “*It grows on the mountains and is even more beautiful in the gardens. The stem is straight, dark green, and 6 feet tall. In the summer it has blue bells, but you can also find it with yellow bells, which is called the dog’s death. Both are poisonous, but in some places it is used for livestock. It is also useful for people with gout, … rheumatic inflammation, ascites, skin diseases, stomach cancer, French fever, … epilepsy, and tuberculosis. It is prepared in various ways like an extract, which is put into pills or solutions. It is used 4 times per day in doses of ½-2 grans; in water for eyes, it is diluted to concentrations of 5-10 grans per one ounce. In addition to the root, there is a small root in the shape of a tooth and it is said that it can help the teeth*”. In the nineteenth century, many manuscripts mentioned Nicolas Lémery in the preface [22, 23]. The reference book was likely “*Dictionaire, ou traité universel des drogues simples”*, in which aconite was included [42].

Medicinal and veterinary use of *Aconitum* species was rarely reported in ethnobotanical studies in Europe. Sarić-Kundalić (2011) mentioned the external use of *Aconitum toxicum* Reich roots for treatment of rheumatism in Bosnia and Herzegovina [43]; Bellia and Pieroni (2015) reported the use of *Aconitum napellus*, which was eaten fresh and used as an abortifacient in cattle [44]. Behxhet Mustafa (2012) reported the use of *Aconitum divergens* Pančić on the Kosovar side of the Albanian Alps; the aerial parts were used in an infusion for stomach disorders, as an antiseptic for oral cavities and as an antihemorrhoidal therapy; the whole plant was prepared as an infusion and used to lower cholesterol levels; and the leaves were squeezed and topically applied to wounds and used as an antibacterial agent for skin infections [45].

This paper reports the results of a study on the folk use of *Aconitum* spp. in Solčavsko, a remote area in northern Slovenia. The study focused on the medicinal and veterinary use of *Aconitum* spp. and aimed to investigate how the plant is or was prepared as medicine, for which indications it is or was used, dosing of the medicine, and whether the folk use of aconite was connected to poisoning incidents.

## Methods

### Research area

The folk use of *Aconitum* spp. was investigated through interviews with local people in Solčavsko, which is an alpine area located in the northwestern part of the Upper Savinja Valley in Slovenia near the border with Austria. Solčavsko comprises three alpine glacial valleys: Logarska dolina, Matkov kot, and Robanov kot. Solčavsko spatially coincides with the Municipality of Solčava. Almost 75% of the municipality falls under the protected areas of Natura 2000. The largest settlement in Solčavsko is Solčava, a village with 204 inhabitants (2016). Solčavsko is 103 km^2^ in area and is surrounded by two mountain ranges, Karavanke and Kamniško-Savnijske Alpe. In 2013, the total population was 516 inhabitants. Solčavsko has a low-mountain climate. The area is covered with snow up to 112 days per year and even longer higher in the mountains. In the village of Solčava, the mean precipitation is 1500 mm per year. Forests cover 80% of the area in Solčavsko; silver fir (*Abies alba*), beech (*Fagus sylvatica*) and spruce (*Picea abies*) are present; toward the tree line, dwarf mountain pine (*Pinus mugo*) and larch (*Larix decidua*) also grow [46].

Until the First World War, Solčavsko was economically more connected to Carinthia, especially to the town of Železna Kapla. People from Solčava traversed a mountain pass to Železna Kapla to sell home products, buy essential food and perform seasonal work. Solčava became connected by road to Lower Styria on the southern side at the end of the nineteenth century. After the First World War, the connection to Carinthia was lost as Solčavsko fell under the jurisdiction of the Kingdom of Yugoslavia and Železna Kapla became part of Austria. In the nineteenth century, people worked in charcoal burning and/or were farmers and produced livestock; at the beginning of the twentieth century, people also earned a living from timber harvesting. Later in the twentieth century, people worked in the wood, textile, and electrical industries. In the present day, many people are still farmers; in 2010, there were 55 farms in Solčavsko, and meat cattle farming was the most common [46].

Solčavsko is a remote area where healthcare has long been underdeveloped. According to a report from 1815, a local priest was of the opinion that the expertise of qualified physicians was not necessary in Solčava because people there did not suffer any illnesses; instead, some people died of old age, and others fell off cliffs (In Slovene: *Tukaj navadno ne poznamo drugih bolezni, nego eni umerjejo vsled visoke starosti, drugi pa raz pečine popadajo*) [47]. People suffering from illness treated themselves or sought help from a folk healer. According to Videčnik (1995), there were many folk healers in the Upper Savinja Valley; an especially well known healer was the previously mentioned Vid Strgar-Fida from Solčava [48]. In 1933, it was recorded that there was no doctor or dentist in Solčava, and sanitary conditions were very poor. Solčava obtained a water supply system only at the end of 1933. There were also women during that time who helped people by walking to distant places, such as Železna Kapla (30 km away), Črna na Koroškem (25 km away) and Kamnik (36 km away), and brought back medicines from a doctor or a pharmacy in exchange for payment. These women were called “potovke” and were present throughout the Upper Savinja Valley. After the Second World War, a doctor came to Solčava once per week [49]. Currently, people from Solčavsko can obtain primary healthcare at the health center in Luče, which is 10 km away from Solčava.

### Interviews

Interviews were conducted from April 2015 to April 2016, and 19 informants in Solčavsko and 3 informants in Luče (Table 1). Additionally, two doctors who worked in this area were interviewed. In the beginning of the study, the informants were selected randomly, while later on, purposive and snowball sampling methods were used in this study [50, 51]. The selection of the informants was based on recommendations from previous informants, who had begun to recommend other people who knew about the use of the herbal medicine. The interviews were conducted in the homes of the informants. During the interviews, the plant was called by its local name “voukuc” for better identification, and a picture of the plant was shown to the informants. The language used in the interviews was Slovenian. The informants were made aware of the scope of the study, and informed written consent was obtained. Semi-structured interviews were used to collect data. Informants were asked the following questions:

- *Do you know of “voukuc”?*
- *Do you know how to use “voukuc”?*
- *Do you remember any cases of aconite poisoning due to “voukuc” or its preparations?*

**Table 1:** Informants’ ages and number

Age (years)	Number of informants in Solčavsko	Number of informants in Luče
Less than 49	4	/
50–59	/	/
60–69	3	1
70–79	6	/
80–89	5	2
90–99	1	/

Interviews were recorded using a voice recorder, and most of them were simultaneously written down in a notebook. To protect their privacy, the informants are identified in the text with letters and the informant’s real age at the time of the interview.

### Ethanolic extracts obtained from the informants

With the informants’ consent, three homemade ethanolic extracts of *Aconitum* spp. were sampled, and the concentration of aconitine was measured.

### Ethanolic extracts prepared in the laboratory

To confirm the authenticity of the reported preparation methods, ethanolic extracts were prepared based on recipes reported by informants. Roots of *Aconitum tauricum* Wulfen were collected on Lanež Mountain in Solčavsko in August 2015. The plants were identified using a dichotomous key, *Mala flora Slovenije* [52], and a voucher specimen (L15100) was stored at the herbarium of the Faculty of Pharmacy, University of Ljubljana.

Two dried roots of *Aconitum tauricum* Wulfen were sliced into pieces, and two extracts were prepared from each root: one with 40% and the other with 70% ethanol (*v/v*). Instead of homemade fruit brandy, 40% ethanol (*v/v*) was used, and 70% ethanol (*v/v*) was used instead of a strong spirit called “cvet”, which contains approx. 70% (*v/v*) ethanol. Altogether, four ethanolic extracts were prepared from two roots. In all the extracts, the ratio of the bulk volume of the sliced roots to the volume of the extract was 1:10. Approximately 5 ml of sliced roots was placed in a measuring cylinder, and ethanol was added to make a total of 50 ml. The exact weight of the roots and added ethanol was measured on a scale. The ethanolic extract (the roots and ethanol) was then transferred to a 50 ml glass tube and incubated in an ultrasonic bath for 20 min at room temperature. The ethanolic extracts were wrapped into aluminum foil and placed in a dark room for maceration; after 5 weeks, they were sampled for analysis. One ml of the extract was placed in a 1.5 ml centrifuge tube and centrifuged at 10000 rpm for 5 min; the sediment was discarded, and the supernatant was stored at −20 °C until analysis.

### LC-MS/MS method for the determination of aconitine in preparations of *Aconitum* spp.

Aconitine (≥95%) was purchased from Sigma (Steinheim, Germany). HPLC-grade acetonitrile Chromasolv® gradient grade for HPLC (>99.9%) was purchased from Sigma-Aldrich (Steinheim, Germany), and formic acid (98–100%) from Kemika (Zagreb, Croatia) and pure water (18 MΩ) was prepared with a Sartorius Stedim Biotech ARIUM pro UV/DI system.

For chromatographic separation, an AT 1100 Series HPLC system (Agilent Technologies, Waldbronn, Germany) consisting of a degasser (G13791), a binary pump (G1312A), a thermostatic column department (G1316A) and an autosampler (G1313A) was used. The compound was detected with a Micromass Quattro Micro™ API triple quadrupole (Waters, UK) after positive ionization with electrospray.

A Zorbax Eclipse XDB-C8 RRHT column (50 mm × 4.6 mm i.d., 1.8 μm) protected by 0.5 μm frit (Agilent, P/N 280959–907) was used for chromatography. The temperature of the column department was set to 40 °C. Different mixtures of solvents A (purified water–formic acid–acetonitrile, 89.9:0.1:10.0, *v/v/v*) and B (formic acid–acetonitrile, 0.1:99.9, *v/v*) were studied to select the final composition of the mobile phase. For all further measurements, a mixture of solvents A and B (65:35, *v/v*) was used at a flow rate of 0.7 ml/min. The injection volume was 10 μl. The retention time of aconitine was 1.7 min. No interference was observed at the time of elution.

A solution of aconitine, prepared at a concentration of 10.0 mg/l in a water–methanol (50:50, *v/v*) mixture, was used for optimization of mass spectrometric conditions by direct infusion into the source. The following general conditions were chosen: the capillary voltage was set to 3.5 kV, the source temperature was 120 °C, desolvation gas nitrogen obtained from a nitrogen generator (Peak Scientific) was heated to 350 °C and delivered at a flow of 600 l/h, the cone gas flow was 60 l/h, and the pressure of the collision gas argon was 50 kPa. Three observed precursor/product transitions (SRM) were *m/z* 646.4 → 586.5 (quantitation trace), *m/z* 646.4 → 526.4, and *m/z* 646.4 → 368.4. The collision energies were 35 eV, 45 eV and 48 eV, respectively. The software used was Waters MassLynx 4.1.

A stock solution of aconitine was prepared in methanol at a concentration of 1 mg/ml and was stored at 4 °C in a dark vial. Seven working standard solutions were prepared by diluting the appropriate amounts of stock solution with the mobile phase ranging from 0.1 to 10.0 mg/l, and preliminary analyses of the samples were performed. A least squares regression analysis was used to generate a calibration curve model. For quantitation, at least 10 data points across the chromatographic peak and signal-to-noise ratio S/*N* > 10 were required. The criterion for limit of detection (LOD) was S/*N* > 3.

The developed method was used in further experiments. To generate a linear calibration curve, a zero sample and 8 calibration standards (0.1, 0.2, 0.5, 1.0, 2.0, 3.0, 4.0 and 5.0 mg/l) were used. The lowest standard was chosen as the limit of quantitation (LOQ); if necessary, it could be lowered further. Three independent calibration curves with duplicates at each level were used for simple validation of method. The observed precision was <10%, the accuracy was within ±11%, and the LOD was 0.001 mg/l. Samples were diluted tenfold with the mobile phase to fall within the range of the calibration curve and were analyzed in duplicate.

## Results and discussion

### General information about the use of *Aconitum* spp.

All the informants in Solčavsko and Luče knew of *Aconitum* spp. and mentioned that they were very poisonous but healing in small amounts. According to the description of the plant, photo recognition, and the reported habitat, the informants most likely used the roots of *Aconitum tauricum* Wulfen and *Aconitum napellus* L. The roots of *Aconitum* spp. were macerated in homemade spirits, and the ethanolic extract was then used to treat various diseases. The informants used the name “voukuc” for both the *Aconitum* plants and the ethanolic extract from the aconite roots. During the interview, four informants showed us their ethanolic extract.

### Collection of the plant

The roots of *Aconitum* spp. were collected from plants growing wild in the mountains and pastures as well as from plants that were cultivated in gardens for easier accessibility. Informant E (woman, 83 years old) mentioned that aconite is most healing if it is collected in the mountains. Some informants mentioned that the thickness of aconite root is important; some preferred thicker roots, and some mentioned that the best roots were young, thin and bright. The informants reported that much aconite previously grew in Solčavsko, but the plant is now rare; the informants believed that this was probably due to excessive wild collection of aconite roots. The informants also reported that people previously collected large amounts of aconite roots and sold them to cooperatives. The collection of aconite roots in exchange for money was also evidenced in newspaper ads that encouraged people to collect different types of herbs for cooperatives, pharmacies and pharmaceutical companies [25–29].

### Preparation of the ethanolic extract

#### Recipes for the preparation of ethanolic extracts

Twelve informants (11 from Solčavsko and 1 from Luče) described the preparation of the ethanolic extract from the roots of *Aconitum* spp. (Tables 2 and 3), and six informants only knew of *Aconitum* spp. and had heard of the ethanolic extract. Altogether, five informants prepared or had prepared the extract and used it themselves; these informants could describe the preparation, dosing and medicinal uses of the ethanolic extract well (Table 2). In addition, seven informants were uncertain when describing the preparation of the extract in detail, as they knew of the extract only through narrations by their parents, grandparents, or acquaintances, or they had observed the preparation of the extract as children (Table 3).

**Table 2:** Preparation of ethanolic extracts from the roots of *Aconitum* spp. and their medicinal use, as reported by 5 informants who knew the preparation well, described it precisely and had prepared it themselves

Informant	Amount of *Aconitum* spp. root	Preparation of the root	Type of solvent	Amount of prepared extract / container	Duration of maceration	Interesting features	Dosage	Medicinal use
I (woman, 78 years old)	Half a bottle of thinly sliced roots	The roots had to be fresh	Very strong spirit; the best was cvet^a^	-Approx. 0.1 L -A small bottle	3 weeks	At the end of maceration, the roots were filtered.	The informant did not remember the exact number of drops	MED: fever
J (woman, 89 years old)	10 cm of sliced roots; the amount of root was approximately one finger high in a 1 L bottle	Clean and freshly collected roots; it does not matter whether you use old or young roots; they used to say that thicker roots were older and stronger and you had to use fewer	Homemade fruit brandy made from pears or plums	-1 L -A bottle	Some time but not long; the roots quickly release the essence	The extract was used for a long time (more than one year); the extract was prepared similarly to other extracts.	A few drops were taken; the informant did not remember the exact number of drops	MED: fever; cold; external use for toothache and inflammation; good for everything; in the war, it was used for pain and inflammation
F (woman, 90 years old)	10 cm of a sliced root that was as thick as a water pipe	Fresh roots collected before the flowering of the plant; roots were collected as soon as the plants started to grow and could be identified; the roots were washed and sliced	Homemade fruit brandy usually made from pears	-1 L - One liter bottle	21 days in a cool place; not in the sun	Everything that remained after the preparation of the extract was well hidden (buried); the extract was used for approximately 2 years.	Adults: 5 to 6 drops Young children: 2 drops The extract was placed on sugar or mixed into water before ingestion	MED: fever, pain (toothache), inflammation
H (woman, 82 years old)	Half a bottle of sliced roots	Washed and sliced roots; thicker roots are older and therefore stronger	Homemade fruit brandy usually made from rowan berries (*Sorbus aucuparia*); strong spirits make the final extract more effective	−0.1 L; it is good that the fruit brandy is at least 1 cm above the sliced roots - A bottle	It is good to leave the extract standing for approximately one month	The extract could be used for 50 years or more.	Adults: 3 to 4 drops Children older than 3 years: 1 drop The extract was placed on sugar or mixed into water before ingestion; milk was consumed with the extract to prevent poisoning Cattle: 10 to 15 drops were placed on bread	MED: pneumonia, influenza, cold, Spanish disease (shaking, fever, nausea, and sore feet) VET: for increased strength in horses
D (woman, 77 years old)	2 small sliced roots	Washed and sliced roots	The best spirit is cvet^a^, but homemade fruit brandy was mostly used	- 0.1 L - A bottle	It is good to leave the extract standing for a short amount of time		Adults: 3 drops Children: 1 drop Horses: 1 drop	MED: pneumonia, pain VET: for increased strength in horses

*MED* medicinal use, *VET* veterinary use

^a^The first flow of the second distillation of fruit brandy

**Table 3:** Preparation of ethanolic extracts from the roots of *Aconitum* spp. and their medicinal use as reported by 7 informants who were somewhat uncertain in describing the preparation; they knew the ethanolic extract from the narration of their parents, grandparents, or acquaintances or they had observed the preparation as children

Informant	Preparation of the extract	Dosage	Use	Interesting features
B (woman, interviewed in Luče, 88 years old)	1 root was macerated in 250 ml of schnapps	Horses: 10 drops on bread	VET: hives and cold in horses	The informant’s husband prepared the extract for horses.
C (man, 64 years old)	Roots were macerated in a large half-liter bottle	Adults: 5–10 drops Children: not more than 3 drops	MED: fever and cough	The informant described the preparation after narration of a woman who was requesting aconite.
K (woman, 78 years old)	2 roots the size of a finger were soaked in 1 l of schnapps	Adults: 3 drops Children: 2 drops	MED: fever	The informant did not prepare the extract by herself
L (woman, 73 years old)	3 cm of a root was soaked in 1 dl of schnapps and placed in a dark room for 3 weeks	Adults: 5 to 7 drops Drops were placed in water. It was not good if drops were placed in tisane	MED: fever, for (physical) strength (chronic use)	The informant did not prepare the extract by herself. Her parents had it at their home.
M (woman, 69 years old)	A root that was long as a little finger was macerated in a large half-liter bottle of schnapps	Adults: 2 to 3 drops Children: a drop less than for adults	MED: fever VET: indigestion in livestock and strength in livestock	In the informant’s childhood, her mother prepared the extract.
N (woman, 75 years old)	1 tablespoon of sliced roots was placed into 2 dl of schnapps	A few drops were placed in water and drunk	MED: fever VET: for weakened animals	The informant did not prepare the extract; her mother advised her to prepare it.
G (woman, 87 years old)	2 dl of roots was placed into a large half-liter bottle of schnapps and left to soak for 14 days	Adults: not more than 5 drops Children: just a small amount	MED: severe illnesses, fever, and inflammation	
A (woman, 82 years old)	3 sliced roots were placed into half a liter of schnapps	Adults: 6 to 8 drops Children: half as much as for adults	MED: fever, various diseases	The last time the informant prepared the extract was in 1946.
E (woman, 83 years old)	2 to 3 roots were placed into half a liter of schnapps; the roots filled almost half the bottle; the soaking time was short	Adults: 9 drops Children: 3 drops Drops were placed in water and drunk	MED: prevention of diseases, fever, influenza, treatment of headache (lubrication of the forehead); “good for everything”	The last time the informant used the extract was 50 to 60 years ago; she prepared it herself.

*MED* medicinal use, *VET* veterinary use

Clean and freshly collected roots of *Aconitum* spp. were used for the ethanolic extracts; the roots were sliced into pieces and soaked in homemade spirits. The reported recipes for the ethanolic extracts varied in the types of spirits used, the ratio of the amount of roots to the spirits, and the final volume of the prepared extract. Some informants reported the use of a homemade fruit brandy (schnapps) from pears, plums or rowan berries (*Sorbus aucuparia*) containing approx. 40% ethanol; others reported the use of a “strong” spirit called “cvet” containing approx. 70% ethanol. “Cvet” is obtained from the first flow of the second distillation of fermented fruits, while fruit brandy is obtained from the second flow of the second distillation. “Cvet” has a burning taste because it contains some methanol; it is not used for drinking and is thus discarded in the distillation process. The amount of the prepared extract varied from 0.1 to 1 L. Some informants were precise in the description of the amount of aconite root used, while others were not. In the reported recipes, the ratio of the volume of the roots to the volume of the prepared extract varied from 1:2 to 1:20. In comparison, the preparation of aconite tincture described in the sixth and seventh editions of the Austrian Pharmacopoeia [9, 10] defined the ratio of the aconite roots to diluted ethanol (*w/w*) as 1:5, while the first five editions of the German Pharmacopoeia prescribed the ratio as 1:10 [15–19].

#### Analysis of homemade ethanolic extracts

To confirm the use of *Aconitum* spp. in homemade ethanolic extracts, three samples of ethanolic extracts were obtained from the informants (a fourth extract was shown to us, but the ethanol had completely evaporated; therefore, we did not sample this extract), and the concentration of aconitine was measured. Two samples contained aconitine, and one did not (Table 4). The highest concentration of aconitine was measured in the extract from informant M. She reported that the extract was approx. 100 years old and that adults could take 2 to 3 drops in case of illness. We can conclude that at this dose, a person would ingest 2 to 3 μg of aconitine, which is approximately 1000- to 3000-fold less than the estimated minimal lethal dose of aconitine (3 to 6 mg) [53]. The second extract was 18 years old, and the concentration of aconitine was approximately 7-fold lower than in the first extract. The third extract had been prepared recently (2015), and the concentration of aconitine was below the limit of detection (<0.001 mg/l); it is most likely that the extract was not prepared with the roots of *Aconitum* spp. The owner of the extract reported that she did not collect the roots herself but obtained them from an acquaintance. Interestingly, she mentioned that her family had already used this extract and that it helped.

**Table 4:** Concentration of aconitine in homemade ethanolic extracts of roots of *Aconitum* spp. obtained from informants

No. of the extract	Informant	Age of the extract	Concentration of aconitine [mg/l]
1.	M (woman, 69 years old)	The extract was approx. 100 years old.	20.0
2.	P (woman, 66 years old)	The extract was prepared in 1998.	2.8
3.	D (woman, 77 years old)	The extract was prepared in 2015.	<0.001
4.	A (woman, 82 years old)	The extract was prepared in 1946.	Not analyzed

#### Analysis of the ethanolic extracts prepared in the laboratory

To confirm the plausibility of the reported preparation methods, four ethanolic extracts were prepared based on the informants’ reports. For the extracts, two dried roots of *Aconitum tauricum* Wulfen, A and B, and two concentrations of ethanol, 40% and 70%, were used. In all four extracts, the ratio of the bulk volume of sliced roots to the final volume of the extract was 1:10. The concentration of aconitine in the laboratory extracts (Table 5) was comparable to the concentration in the informants’ extracts; therefore, we can conclude that the reported preparation methods were genuine. The slight variations in the concentration of aconitine in the extracts were likely due to inhomogeneity of the plant material. The concentration of ethanol was unlikely to influence the amount of aconitine extracted from the roots of *A. tauricum*.

**Table 5:** Concentration of aconitine in ethanolic extracts prepared in laboratory based on informants’ reports

Ethanolic extract	Root sample	Concentration of ethanol	Weight of the dry root [g]	Volume of the ethanol [ml]	Concentration of aconitine [mg/l]
1	A	40%	3.025	46.20	19.1
2	A	70%	3.015	45.88	17.3
3	B	40%	3.009	45.89	9.8
4	B	70%	3.023	45.68	15.3

### Dosing

The ethanolic extract was administered in drops. Placing a finger on the neck of the bottle caused drops to form, and a certain number of them were placed on a spoon; the extract was then mixed with water or placed on sugar for ingestion. Some informants mentioned that the extract would lose effectiveness if it were mixed into a tisane. Each informant reported a different number of drops; interestingly, many informants mentioned that it is better to take a drop less than a drop too much and that a drop too much could be fatal. From the informants’ reports, we can observe that the number of administered drops was higher for adults than for children; the number of drops for adults varied from 2 to 10 and for children from 1 to 3 (Tables 2 and 3). The ethanolic extract was also used in animals, and there were three reports of dosing. In two reports, the number of drops was higher than for adults (Table 2, informant H; Table 3, informant B), and in one report, the number of drops was lower than for adults (Table 2, informant D). We would expect that informants who prepared stronger extracts would use smaller doses and vice versa, but the ratio of the amount of root to the amount of spirits used for the extract did not correlate with the dosing of the extract.

It is likely that there were no rules for dosing of the extract, and the number of drops depended on self-estimation of the nature and quality of the prepared extract. We conducted two interviews with informant H (woman, 82 years old) over a period of four weeks, and in each she reported a different number of drops used. We asked her whether there were any rules for dosing, and she replied that each person knew how many drops of the extract should be used for different illnesses. Although not reported, it is possible that the person who prepared the extract also tested it drop by drop before the entire family used it.

### Folk medicinal use of *Aconitum* spp.

The informants reported many indications for the ethanolic extract of *Aconitum* spp. The extract was used internally for fever, pain, general stimulation of the body, influenza, pneumonia, cough, sore throat, cold, and Spanish disease (shaking, fever, nausea, and sore feet). The extract was also used externally on inflamed areas on the skin, on aching teeth and on the forehead to treat headache. Bohinc (1992) mentioned that doctors prescribed aconite for some of the above-mentioned indications, such as fever, inflammation of the respiratory tract, and toothache. It is possible that some people also used the roots of *Aconitum* spp. for cancer treatment. Informant R reported that during his work as a shepherd on a mountain, he fulfilled the wish of a man who had cancer. He collected roots of *Aconitum* spp. for him, and this man chewed the roots; supposedly, his mouth became numb. Two informants reported that they knew of people who used the extract every day for increased (physical) strength.

Many informants mentioned that the ethanolic extract was used for everything. Informant J (woman, 89 years old) reported that the extract was the main medicine for the inhabitants of Solčavsko before the war and was used for everything; no one even considered calling a doctor. The locals also used it to relieve pain and in various inflammations during the Second World War. Informant H (woman, 82 years old) reported that there used to be a rule that every house should have “voukuc”, juniper oil, arnica, and black oil (black oil was prepared from pine resin). She also mentioned that the extract helped immediately with various illnesses. Informant E (woman, 83 years old) mentioned that the former use of the extract was comparable to the present-day use of Lekadol (brand name for paracetamol). Informant J (woman, 89 years old) also reported that the extract helped immediately with various conditions (health problems); there was a folk saying: “Kar voukuca mu dej” (in English: you should give him aconite).

### Folk veterinary use of *Aconitum* spp.

Informants also mentioned that roots of *Aconitum* spp. were used to improve the health of pigs. Informant B (woman, 88 years old) reported that her neighbor had the roots of *Aconitum* spp. built into the feed trough; the roots were then extracted into the pig feed through small holes. The neighbor claimed that his pigs were very healthy and tried to persuade the informant to use such feed troughs for her pigs as well, but she did not dare. Informant A (woman, 82 years old) reported previously wrapping the roots of *Aconitum* spp. in socks and dipping them into the pig feed to prevent various diseases. Möderndorfer (1964) also reported that aconite roots were built into feed troughs for pigs in the beginning of the twentieth century [23].

Ethanolic extract of *Aconitum* spp. roots was used for the treatment of heaves, cold, and indigestion and for increased strength in horses. The extract was administered orally; a certain number of drops were placed on a piece of bread or on lard. Furmans (horse and cart drivers) used to give the extract to horses for better recovery (personal communication with the local innkeeper in Solčavsko, 2015). Informant H (woman, 82 years old) reported that her grandfather bought very weak horses at a low price and then gave them 10 to 15 drops of the extract every day for two months; after three months, the horses were strong and beautiful. Informant M (woman, 69 years old) also reported that the ethanolic extract was used for indigestion and increased strength in livestock.

### Storage of the ethanolic extract of *Aconitum* spp.

Some informants reported that significant attention was paid to the storage of the ethanolic extract of *Aconitum* spp., and others reported that the extract was stored among other bottles. Informant O (woman, 86 years old) mentioned that as a child she was very afraid of the pantry in which the extract was stored; she believed that her mother kept viper oil inside the pantry. The informant explained that her mother probably frightened the children with viper oil to keep them away from the pantry. Informant C (man, 64 years old) reported that only his mother and grandmother knew where the extract was hidden. Similarly, Informant K (woman, 87 years old) reported that her parents hid the ethanolic extract in the cupboard, which was built into a wall and always locked.

### Poisonings with *Aconitum* spp.

A local physician (man, 67 years) and a nurse (informant F, woman, 90 years) who worked in this area between 1958 and 2010 did not report any intervention due to poisoning with *Aconitum* spp.; however, other informants reported 9 cases of poisoning with the plant (Table 6). In these situations, people had helped themselves, e.g., many informants reported that poisonings with *Aconitum* spp. were treated with large quantities of milk, which induced vomiting. The use of milk was also reported for poisoning with deadly nightshade (*Atropa belladonna*). Informant G (woman, 87 years old) reported that her brother was given milk to drink when he was poisoned with *A. belladonna*; in addition, his mother gave him two drops of ethanolic extract of aconite roots. Sometimes, milk was routinely used together with aconite as a detoxifier; informant H (woman, 82 years old) reported that after a few of drops of the ethanolic extract had been taken, some milk was drunk. The informant explained that the milk would prevent poisoning if the dosage were accidentally excessive.

**Table 6:** Informants’ descriptions of poisonings with *Aconitum* spp.

Informant’s description		Cause of the poisoning
1.	My father was very hot, and as usual he wanted to drink a little bit of cognac to not catch a cold. He confused cognac with voukuc^a^. (Informant J, woman, 89 years old)	Voukuc^a^ was mistaken for cognac.
2.	My mother often drank a little bit of homemade fruit brandy. But one time she confused the fruit brandy with voukuc^a^. She felt better when she was outside and walking, although the children had to assist her with walking. The problems lasted for approximately three hours. (Informant O: woman, 86 years old)	Voukuc^a^ was mistaken for fruit brandy.
3.	At the end of work on the farm, some old women gave a drink to a boy who helped them, but the women had mistaken fruit brandy for voukuc^a^, and the boy died. (Informant M: woman, 69 years old) (The informant reported this case after narration by her sister.)	Voukuc^a^ was mistaken for fruit brandy.
4.	My husband’s grandmother gave some homemade fruit brandy to workers at the end of the work on the farm. When she was pouring the last glass, a small root of aconite/voukuc^a^ came out of the bottle. She immediately gave everybody milk to drink, and because of fear, she moved to some small cottage for a few days. Fortunately, all the workers survived. (Informant P: woman, 66 years old; and informant A: woman, 82 years old)	Voukuc^a^ was mistaken for fruit brandy.
5.	When I was ill, my younger sister brought me a medicine to drink. I started to suffocate and called my mother. She immediately realized that I was poisoned with voukuc^a^ and gave me milk to drink until I threw up everything. (Informant A: woman, 82 years old)	Voukuc^a^ was not protected from the reach of children.
6.	My brother and his friend were collecting medicinal plants in the wild, and suddenly something stung my brother’s neck. He scratched his neck with his hands, but he had collected aconite with them previously. In this way, he poisoned his blood and developed bulging eyes, a red neck, and blisters under his arms. The doctor gave him injections against the poisoning, and he got better.^b^ (Informant H: woman, 82 years old)	This was most likely not a poisoning with *Aconitum* spp. but an allergic reaction.
7.	My brother cleaned and prepared aconite roots for voukuc^a^ but accidentally left a small piece of aconite on the floor in front of the house. At that time, we also had released pigs outside, and one of them ate the root. The pig started to salivate, and we gave him milk to drink. (Informant B: woman, 88 years old)	Carelessness in cleaning up the workplace
8.	My father told me that a farmhand once committed suicide by drinking a spoonful of “voukuc”. He died instantly. (Informant D, woman, 77 years old)	Intentional poisoning - suicide
9.	A woman had a fight with her husband, and then she drank voukuc^a^. The woman got very weak and started foaming at the mouth.She survived because people gave her milk to drink until she threw up everything. (Informant I: woman, 78 years old; and informant H: woman, 82 years old)	Intentional poisoning - a suicide attempt

^a^Both *Aconitum* spp. plants and the ethanolic extract from roots of *Aconitum* spp. are called voukuc in Solčavsko

^b^The informant probably reported an allergic reaction and not a poisoning with *Aconitum* spp.

Informants reported four poisonings in which an alcoholic drink (homemade fruit brandy or cognac) was confused with the ethanolic extract of *Aconitum* spp. These accidents probably occurred because the bottle of the extract was improperly labeled and stored among other bottles. One poisoning also occurred because the extract was stored within reach of children. Informants reported two cases in which the extract was used as a means of suicide. The informants did not describe any poisoning due to improper dosing of the extract, although the recipes and dosages differed between the informants’ reports. It is likely that this was due to the use of small doses of the extract, which is evident from the report by informant M; she reported a dose for an adult that was 1000-fold lower than the estimated lethal dose (her extract contained 20.0 mg of aconitine/l). It is also possible that poisonings due to improper dosing were withheld from the interviewer. A review of poisoning incidents traced to *Aconitum* spp. that occurred between 1958 and 2013 and had been registered with the Poison Control Centre of Ljubljana University Medical Centre showed that there were no reported poisonings due to medicinal use of *Aconitum* spp.; the most frequent cause of poisoning with *Aconitum* spp. was confusion non-toxic herbs with *Aconitum* spp.

### Views of medical practitioners on the folk use of *Aconitum* spp.

Two local physicians and a nurse (informant F) were interviewed about their views on the folk use of *Aconitum* spp. All three medical practitioners were retired at the time of the interview. The first local physician (man, 67 years old) reported that *Aconitum* spp. was slightly mysterious. His patients never told him that they used *Aconitum* spp., but he had heard rumors that people prepared ethanolic extracts from the roots of this plant and called it “voukuc”. He had also heard a few times that the extract stimulates and increases performance, but he never thought that it was used as a medicine. He mentioned that traditional medicine was neglected during his medical studies (1968–1975) and that there was excessive confidence in science. During his studies and career, he did not hear anything official about *Aconitum* spp. The second physician (man, 88 years old) had worked in Solčavsko for several years. He reported that he did not know anything about the medicinal use of *Aconitum* spp., but he emphasized that people from Solčavsko sought medical help only for illnesses that they could not treat themselves, such as heart problems and problems with blood pressure. In contrast, the nurse (informant F: woman, 90 years old) reported that she knew of the extract from childhood. Her patients had also told her that they used the extract from *Aconitum* spp. but were afraid to inform the doctor of this. She believed that her patients trusted her more because she was a local, they knew her well, and she was a nurse. She told patients who used *Aconitum spp.* as a medicine that this plant was an old medicine and that the new medicines are more effective and are not as dangerous.

Since neither physician knew how *Aconitum* spp. was used among people, it is likely that patients hid its use from them. Informant H (woman, 82 years old) reported that she prepared the ethanolic extract from roots of *Aconitum* spp. and gave a few drops to her two children when they were seriously ill. When she told this to the physician, he was somewhat angry with her. He told her that one becomes resistant after taking the extract, which is very dangerous in other illnesses. She did not dare to use the extract again but kept it just in case. It is likely that other people quickly heard of such bad experiences with a physician and became cautious in the transmission of such information. In contrast, patients told the local nurse that they used *Aconitum* spp., probably because they found her to be trustworthy.

### Existence, preservation and loss of medicinal use of *Aconitum* spp.

The exact origin of the present folk use of aconite roots for medicinal purposes in Solčavsko is unclear, but it is known that aconite roots were present in the sixth and seventh editions of the Austrian Pharmacopoeia, which were official in the Slovenian territory from 1869 to 1906. All earlier editions of the Austrian Pharmacopoeia contained aconite herb. Medicinal use of aconite was also described in the manuscript of Vid Strgar-Fida, a folk healer from Solčava. In a manuscript written in 1866, he did not mention which part of the plant was used for medicinal purposes, but the German name for the plant, “eisenhutkraut”, indicates that it is likely that aconite herb was used (“eisenhut” means aconite, and “kraut” means herb). Vid Strgar wrote the manuscript in 1866 and died in 1922; it is therefore possible that he learned that pharmacists prepare medicines from aconite roots later in his life. It is also possible that Vid Strgar taught people in Solčavsko how to prepare and use the ethanolic extract from aconite roots.

Once the medicinal use of aconite was established, elements of continuation and discontinuation influenced the medicinal use of aconite in Solčavsko. The accessibility of aconite roots was a basic element in the formation and preservation of folk medicinal use of aconite in Solčavsko. From newspaper ads, it is evident that people were encouraged to collect aconite roots in exchange for money, even when the plant was not an official medicine. Additionally, informants reported that people collected large amounts of aconite roots and sold them to cooperatives. Although not reported by the informants, gathering of aconite roots for money could also represent evidence to some people that *Aconitum* spp. was an important and valuable plant; the financial value of the plant could also strengthen their belief in its healing properties. The remoteness of Solčavsko, difficulty of access to neighboring areas and slow economic development also propagated a poor healthcare system and the continuation of old methods for the treatment of diseases.

Better healthcare, awareness of the toxicity of aconite and access to new medicines (in the second half of the twentieth century) were likely the main reasons for the decrease in the medicinal use of *Aconitum* spp. in Solčavsko. Informant J mentioned that she became more cautious when physicians and more educated people began to stress the toxicity of the plant and promote less toxic medicines with similar functions. She said she began to fear the effects of the plant and observed that the use of the plant began to diminish among others for the same reason. Informant L (woman, 73 years old) remembered that after the 1970s, the use of “voukuc” was no longer mentioned. She believed that the use of the extract declined with the beginning of the use of antibiotics. As mentioned above, the local nurse tried to persuade patients to use new medicines that were not as dangerous.

*Aconitum* spp. is no longer used to the same extent as in the past. Only four informants had an ethanolic extract of aconite roots at home, and they reported keeping it in case of need; however, two extracts were prepared more recently (in 1998 and 2015), which indicates that this tradition is still alive. However, it is also possible that some informants did not want to report the present use of the plant. Informant A (woman, 82 years old) believed that preparations of *Aconitum* spp. were still in use, but people did not want to admit it because the plant is protected. Among *Aconitum* species, only *Aconitum anthora*, which grows in the coastal region of southwestern Slovenia [54], is on the red list of Slovenian endangered species [55].

## Conclusions

This study researched the folk use of *Aconitum* species, which were part of the official and folk medicine of the Slovenian territory in the nineteenth century. Due to its toxicity, the plant stopped being used as official medicine in the twentieth century. However, in some areas such as Solčavsko, Slovenia, folk knowledge of the medicinal use of *Aconitum* spp. is still present, although the plant is used much less frequently than in the past. The informants reported the preparation of ethanolic extract made from homemade spirits and aconite roots, most likely from the species *Aconitum tauricum* and *Aconitum napellus*; the use of aconite herb was not reported. The extract was used internally and externally for various indications; use in animals was also reported. There was no general rule for dosing of the extract, since each informant reported a different number of administered drops; however, in all reports, the number of drops was higher for adults than for children. Although recipes for the extract and reported dosages differed greatly among the informants, no poisonings due to the medicinal use of the extract were reported by the informants or by a doctor and a nurse working in that area from 1958 to 2010.

## Abbreviations

DAB: Deutsches Arzneibuch
HPLC: High-performance liquid chromatography
LC-MS/MS: Liquid chromatography tandem-mass spectrometry
LOD: Limit of detection
LOQ: Limit of quantification
MED: Medicinal use
VET: Veterinary use
